# Design and optimization of broadband metamaterial absorber based on manganese for visible applications

**DOI:** 10.1038/s41598-023-38263-x

**Published:** 2023-07-24

**Authors:** Shimaa I. Sayed, K. R. Mahmoud, Roaa I. Mubarak

**Affiliations:** 1 Electronics and Communications Department, Faculty of Engineering, Helwan University, Cairo, Egypt; 2 National Telecommunications Regulatory Authority (NTRA), Giza, Egypt

**Keywords:** Electrical and electronic engineering, Metamaterials

## Abstract

Metamaterial absorbers have been extensively researched due to their potential applications in photonics. This paper presents a highly efficient Broadband Metamaterial Absorber (BMA) based on a Manganese–Silica–Manganese three layer structure with a shaped pattern at the top layer. For maximum absorption efficiency, the geometrical parameters of the proposed absorber have been optimized based on Particle Swarm Optimization (PSO). The optimal structure with a thickness of 190 nm, can achieve more than 94% absorption spanning visible band (400–800) nm with 98.72% average absorption, and more than 90% absorption over the range from 365 to 888 nm. In the range from 447 to 717 nm, the design presented above 99% absorptivity, providing an ultra-wide bandwidth of 270 nm. The physical mechanism of absorption is illustrated through the exploration of the electric and magnetic field distributions. Additionally, the proposed structure maintains 85% absorption stability for wide incident angles up to 70° for both the TE and TM polarizations under oblique incidence. Further, the optimized absorber structure with excellent absorption capabilities makes it suitable for various applications, including optical sensors, thermal emitters, and color imaging applications.

## Introduction

In the last decade, there has been a lot of interest in Metamaterial Absorbers (MAs), which are built with subwavelength-sized unit cells made of Metal–Insulator–Metal (MIM)^1,2^. The distinctive Electromagnetic (EM) properties of metamaterials, such as their negative permeability and negative dielectric constant^3,4^, make it possible to be efficiently applied to the various applications, such as solar energy harvesting^5^, wireless communications^6^, and sensors^7^. Extensive research based on MA designs has been published. Depending on the spectrum range in which the EM metamaterial operates, it may be easily operated for different frequencies, including terahertz^8^, visible, and infrared (IR) regimes^9–11^. For absorption bandwidth classification, narrowband MAs find applications in thermal emission manipulation, sensors, nano-antennas, and resonators^12,13^. Wideband absorbers, on the other side, have uses in thermal emitters, solar energy converters, and a variety of other optoelectronic applications^14,15^.

There has been a wide range of research activities in recent years that broaden absorption bandwidth to improve performance and increase capabilities. The first approach for achieving broadband absorption is to use multi-resonances by integrating various sizes of multiple resonators to form an absorber unit cell. Such absorbers offer high flexibility in achieving the desired absorption spectral properties by varying the geometry and structural dimensions of the resonators involved^16–18^. The second approach is to use multilayer structures with different geometrical parameters in the vertical direction, separated by dielectric layers, to broaden the absorption spectral bandwidth^19,20^. However, adding more layers entails intricate microfabrication processes and increased costs. This may obstruct the advancement of metamaterial absorbers. Consequently, it is essential to create simple topology metamaterial capable of achieving high-efficiency absorption^21,22^.

The MIM configuration can provide enhancement in absorption bandwidth. Up to date, several studies have been proposed to maximize absorption of MA structures in both intensity and broadband^10,23^. The most common method is optimizing the structure dimensions and shaping the top surface metallic layer of the metamaterial structure. For instance, a triangular prism shape metamaterial absorber with an average absorbance of 97.85% had achieved near perfectly absorption in the range of 200 to 2980 nm^24^. Another MA structure with a double sized axe-shaped resonator demonstrated more than 90% absorption in the visible to near-IR spectral range (i.e., from 320 to 982 nm)^25^. In addition, Majid Aalizadeh introduced metamaterial design based on a nanodisk-shaped resonator for light absorption spanning the visible to mid-infrared range (i.e., from 478 to 3278 nm), resulting in a wide band absorption^26^.

For the visible regime, many designs based on MIM configuration have been investigated. With the context, Lai et al.^27^ proposed a tri-layer MA based on Al–SiO_2_ with an average absorptivity above 95% from 450 to 600 nm range. However, the wavelength band is still insufficient to fulfil the rising demands for applications such as solar energy harvesting, which require ultra-broadband with perfect absorption characteristics. Moreover, polarization and oblique incidence sensitivity are not considered due to asymmetry structure. Sultan et al.^28^ investigated a star shaped resonator tri-layer MA with above 90% absorptivity from 389 to 697 nm and up to 60° incidence angle stability. The x and y dimensions of this structure are quite large. Bilal et al.^29^ introduced ultrathin broadband absorber comprised of tungsten nanowires with an absorption level of more than 80% and a band span of 400–750 nm. However, absorbers covering the entire visible spectrum with greater than 90% absorptivity are required.

A thin, broadband visible absorber with perfect absorption and a wide angle of incidence is the optimal design. In this paper, a novel absorber using Manganese (Mn) is designed, providing ultrahigh absorption for the entire visible range spectrum (400–800 nm). The proposed absorber structure is constructed based on MIM configuration, with a square disk surrounded by a square ring-shaped of Mn top layer and SiO_2_ dielectric spacer. To achieve the best performance of the proposed MA design, the Particle Swarm Optimization (PSO) algorithm is used to fine-tune the geometrical parameters of the absorber unit cell. In addition, the electric and magnetic field distributions are discussed to clarify the physical mechanisms underlying broadband perfect absorption.

## Absorber design and optimization

**Figure 1:**
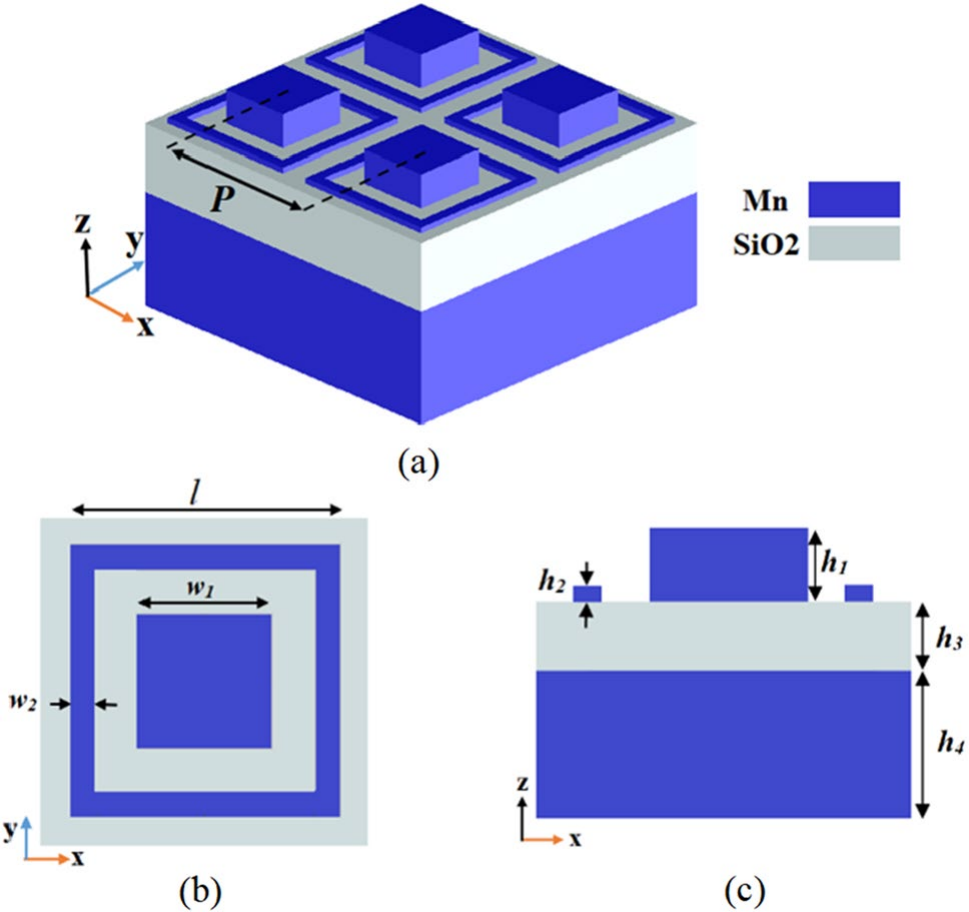
(**a**) Schematic representation of a periodic array of the proposed broadband metamaterial absorber. (**b**) Top-view of a single absorber unit cell. (**c**) Side-view at the center of the absorber unit cell, which all are extracted from Lumerical FDTD solutions software^30^.

**Figure 2:**
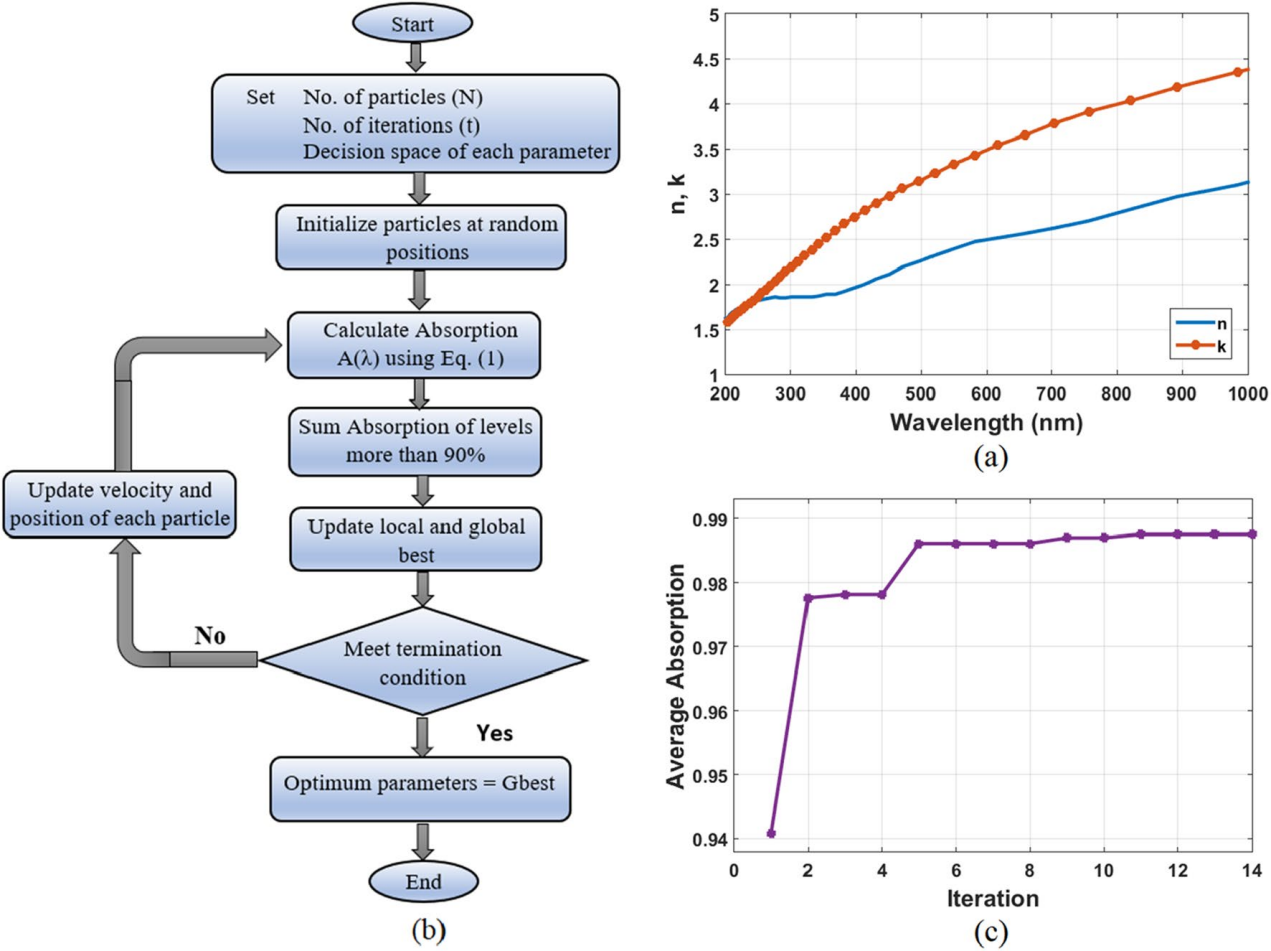
(**a**) Complex refractive index of Manganese (n and k), (**b**) Flow chart diagram of the applied PSO algorithm and (**c**) The average absorption with number of iterations of the applied PSO algorithm.

The schematic representation of the proposed Broadband Metamaterial Absorber (BMA) is depicted in Fig. 1 which is obtained from the Lumerical FDTD solutions software^30^. The top most layer of a square disk surrounded by a square ring-shaped Mn acts as a resonator. SiO_2_ dielectric material is used to separate the top resonator from the bottom Mn layer.This spacer can assist in the construction of Fabry–Perot cavities and can also induce electromagnetic coupling between the top resonator and the bottom Mn layer. The reason for choosing Mn as the metal layer is its real part of permittivity, which varies slowly and covers the visible band. This makes it possible for the structure to be matched to the impedance of free space, leading to the strong penetration of the field^22,31^. It also exhibits strong absorption due to a large imaginary part of permittivity. In addition, Mn has the advantage of being less expensive than metals such as gold, which have been used in various patterned broadband absorbers^22^. It is also very advantageous to use low-cost materials to achieve compatibility for mass production. Figure 2a displays the effective refractive index (n) and the wave vector (k) of the complex refractive index of Mn, which are taken from Johnson and Christy's refractive index database^32^. SiO_2_ is employed as a dielectric material because its relative permittivity is low in the optical wavelength range^11^. The Palik model is used to represent the refractive index of SiO_2_^33^.

The proposed absorber structure consists of three layers Mn–SiO_2_–Mn with a periodicity (*P*) of 300 nm. The top square disk has a width of *w*_1_, and the square ring has a length (*l*) with a width (*w*_2_). The thicknesses of each layer from top to bottom are *h*_1_ and *h*_2_ for the top layer, *h*_3_ and *h*_4_ for the dielectric and substrate, respectively. The thickness of Mn substrate is selected to be greater than the skin depth $\delta \left(\lambda \right)$ to which waves can penetrate. Equation (1) shows how the skin depth of a conductor is calculated^34^. where λ is the wavelength (m), ρ is the resistivity of a conductor (Ω m), *μ* is the permeability (H/m), and C is the speed of light in free space (m/s). For the electrical resistivity of Mn ρ = 144.2 × 10^−8^ Ω m, the skin depth at ranges from a low of 22 nm at wavelength of 400 nm to a high of 31.2 nm wavelength of 800 nm^35^. So, the Mn substrate having a thickness of 100 nm performs as a perfect reflector, resulting in structure transmission is approximately zero.

1 $$\delta \left(\lambda \right)=\sqrt{\frac{\lambda \rho }{\pi \mu C}}$$

As known, The MA structures of different metal and dielectric layer thicknesses operate with varies range of frequencies. The thickness is a primary constraint in realizing a perfect absorber. With an aim to maximize the absorption more than 90% as well as minimize the absorber thickness, PSO algorithm is used for achieving the optimum solution to this problem. The geometrical parameters of the proposed design are suitably optimized for broadband perfect absorption and minimum absorber thickness.

The Finite Difference Time Domain (FDTD) approach is used to numerically model the absorption for our proposed structure, and the PSO algorithm is used to optimize the geometrical parameters. For FDTD simulations, Perfectly Matching Layers (PML) are utilized along the z direction, whereas Periodic boundary conditions are employed for the x and y directions. Mesh step settings are 5 nm, 5 nm and 1 nm in x, y and z-plane respectively. The parameters included in the optimization process are *h*_1_,* h*_2_,* h*_3_,* w*_1_ and *w*_2_. The other parameters which are *l* and *h*_4_ are fixed through the optimization process with 250 nm, 100 nm respectively. Figure 2b illustrates the basic steps of the applied PSO algorithm with a flowchart diagram. After defining the number of particles, number of iterations, and search space boundaries, the PSO algorithm begins by randomly generating initial particles within a specified range. Each particle stands for a possible solution to solve the optimization problem. Next, the absorption of each particle was then calculated using the FDTD method at wavelengths ranging from 400 to 800 nm according to Eq. (2), where A(λ) and R(λ) are absorption and reflection as a function of wavelength, respectively. The objective function is defined by summing absorption of levels more than 90% in 400–800 nm region. The optimization process aims to enhance the fitness value of the objective function to its highest potential.

2 $$A\left(\lambda \right)=1-R\left(\lambda \right)$$

The number of particles (N) and iterations (t) were set to 20 and 50 respectively. At each iteration, the maximum fitness value is identified as the local best. In case the new local best surpasses the global best, the global best is then updated with the new local best. When the number of iterations reached its limit, the optimization process came to an end. If not, the velocity of the particles and their new positions will be updated. The process of optimization was then repeated until the optimal geometrical parameters were attained. The average absorption of the proposed structure per iteration is illustrated in Fig. 2c. As shown, the average absorption remains constant for iteration number 12 and up. Consequently, the results of iteration number 12 are chosen as the geometrical parameters. According to the actual preparation process of geometrical parameters, we approximated their values to real integer numbers. For each parameter, the setting range is stated in Table 1 with its optimum value.

**Table 1:** List of geometrical parameters applied to PSO algorithm for the proposed absorber.

Parameter	Decision space (nm)		Optimum value
	From	To	
*h*_1_	3	40	30
*h*_2_	3	40	5
*h*_3_	40	80	60
*w*_1_	50	150	125
*w*_2_	10	50	20

## Results and discussion

**Figure 3:**
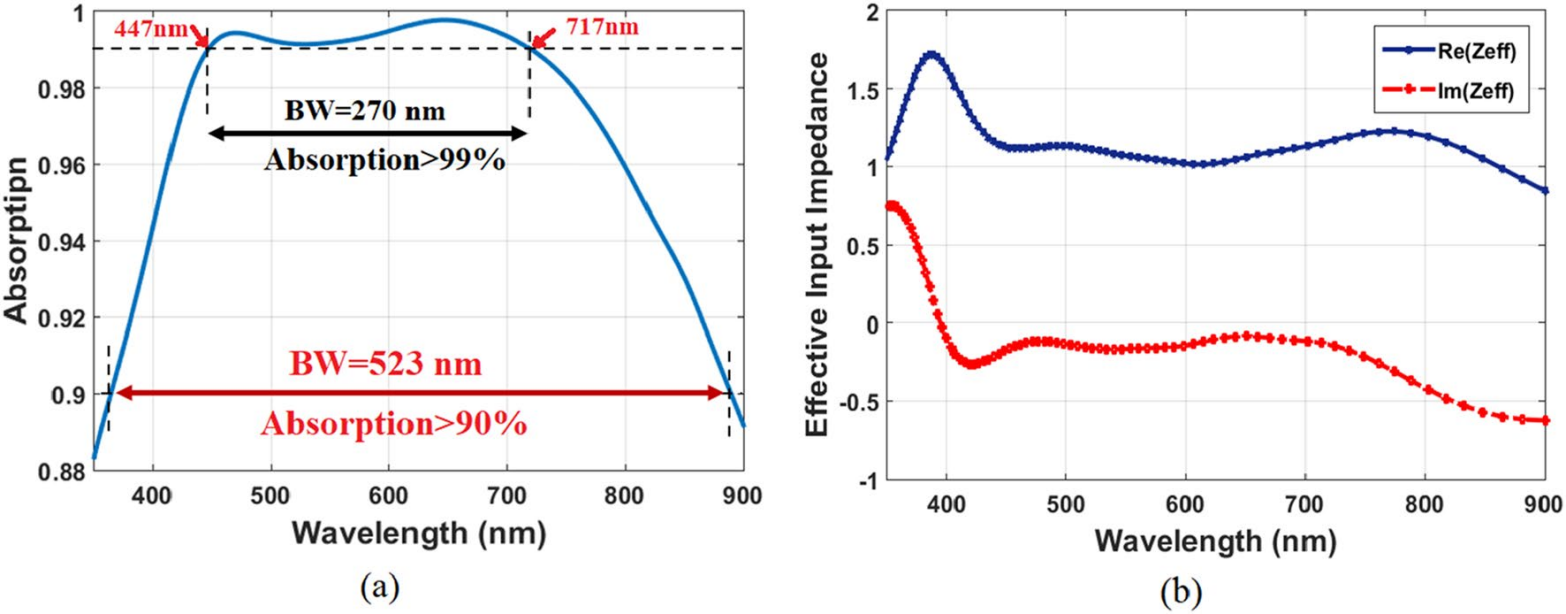
(**a**) Absorption of the proposed absorber design with the optimized parameters and (**b**) Effective input impedance of the absorber design.

In this section, the advantages of absorption characteristics of the proposed MA structure are investigated. After the optimum geometrical parameters have been applied, the absorption spectrum under TE-polarized light is presented in Fig. 3a. The structure exhibits a strong absorption of more than 94% over a broad wavelength range, beginning at 400 nm and going up to 800 nm, and an absorption of more than 90% over the range from 365 to 888 nm. Using Eq. (3), we can figure out that the average absorption reaches up to 98.72% over the band of 400–800 nm. Perfect absorption (over 99%) is achieved from 447 to 717 nm with a bandwidth of 270 nm, and peak absorption is obtained up to 99.8% at the wavelength of 655 nm. Therefore, our results show that the proposed design with the optimized parameters yields great performance.

3 $${A}_{avg}={\int }_{{\lambda }_{1}=400\mathrm{nm}}^{{\lambda }_{2}=800\mathrm{nm}}\frac{A(\lambda )d\lambda }{{\lambda }_{2}-{\lambda }_{1}}$$

The reason behind ultra-high absorption of the proposed structure is that its impedance matched with that of free space. If the scattering parameters (S-parameters) are known, it can be easily to attain a theoretical observation about this remarking. The scattering parameters for reflection and transmission are S_11_ and S_21_, respectively. S_21_ can be reasonably equal to zero owing to the presence of the thick metallic bottom layer. The effective input impedance (Z_eff_) of our proposed absorber can be obtained from S-parameters using Eq. (4) ^36,37^. Figure 3b displays the simulation results of the normalized Z_eff_ for the pattern shaped Mn absorber. It can be clearly observed that real component of the structure impedance is close to 1, while the imaginary component is close to 0 through the visible regime (400–800 nm). This means that impedance of the pattern-shaped Mn absorber closely matches free space impedance.

4 $${Z}_{eff}=\sqrt{\frac{{(1+{S}_{11})}^{2}-{S}_{21}^{2}}{{(1-{S}_{11})}^{2}-{S}_{21}^{2}}}=\frac{1+{S}_{11}}{1-{S}_{11}}$$

**Figure 4:**
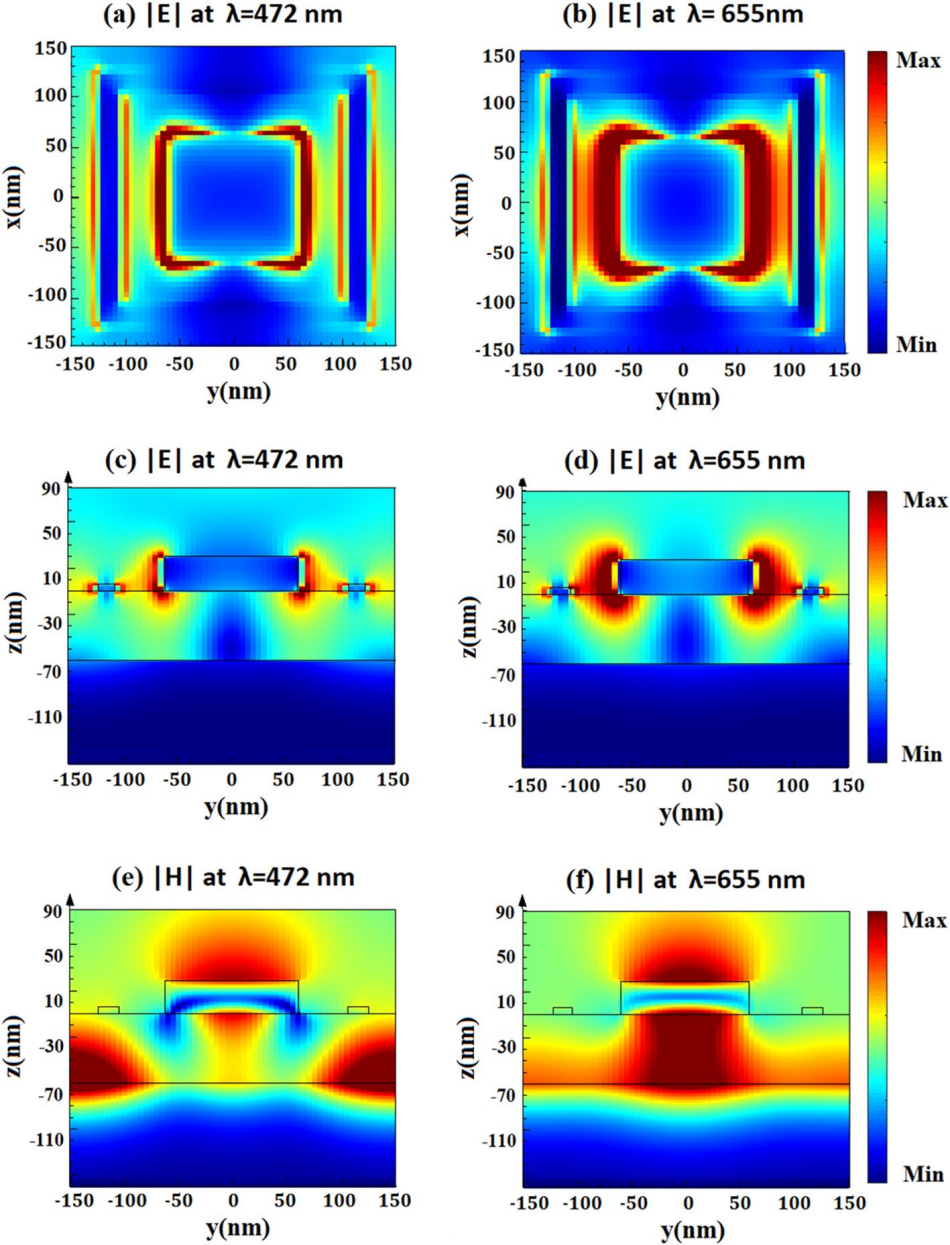
Electric field distributions (**a**,**b**) in the x–y plane and electric field distributions (**c**,**d**) in the z–y plane, and magnetic field distributions (**e**,**f**) of the proposed unit cell at different resonant wavelength peaks (472 nm, and 655 nm), respectively.

To further illustrate why the proposed MA structure has broadband and perfect absorption, distributions of electric and magnetic fields (|E| and |H|) are simulated and shown in Fig. 4. Vertically TE-polarized waves of two resonant wavelength peaks, at 472 nm and 655 nm, are incident on the structure. Electrical distributions in the x–y plane are depicted in Fig. 4a,b, and electrical distributions in the z–y plane are depicted in Fig. 4c,d, respectively. The electric field is concentrated in the region between the square disk and square ring metals, and also focused around the metal edges of Mn. As a result, these distributions strongly suggest that Surface Plasmon Polaritons (SPP) are excited inside the structure^37^. However, the magnetic field distributions are clearly different. Specifically, at 472 nm seen in Fig. 4e, it is believed that the resonance is Propagating Surface Plasmon resonance (PSP). At the resonant wavelength of 472 nm, the magnetic field accumulated in the square disk Mn metallic top layer and SiO_2_ dielectric spacer, and it spread through the adjacent cells. This demonstrates that PSP resonance is responsible for the absorption around 472 nm^5,25^. Figure 4f shows that, at a wavelength of 655 nm, the magnetic field is strongly localized within the space between the top square disk of Mn and the Mn bottom layer. This confirms that the Localized Surface Plasmon resonance (LSP) has been excited^25,38^. The magnetic field distributions shown in Fig. 4e,f confirm the excitation of PSP and LSP resonances, which further widens the absorption bandwidth.

**Figure 5:**
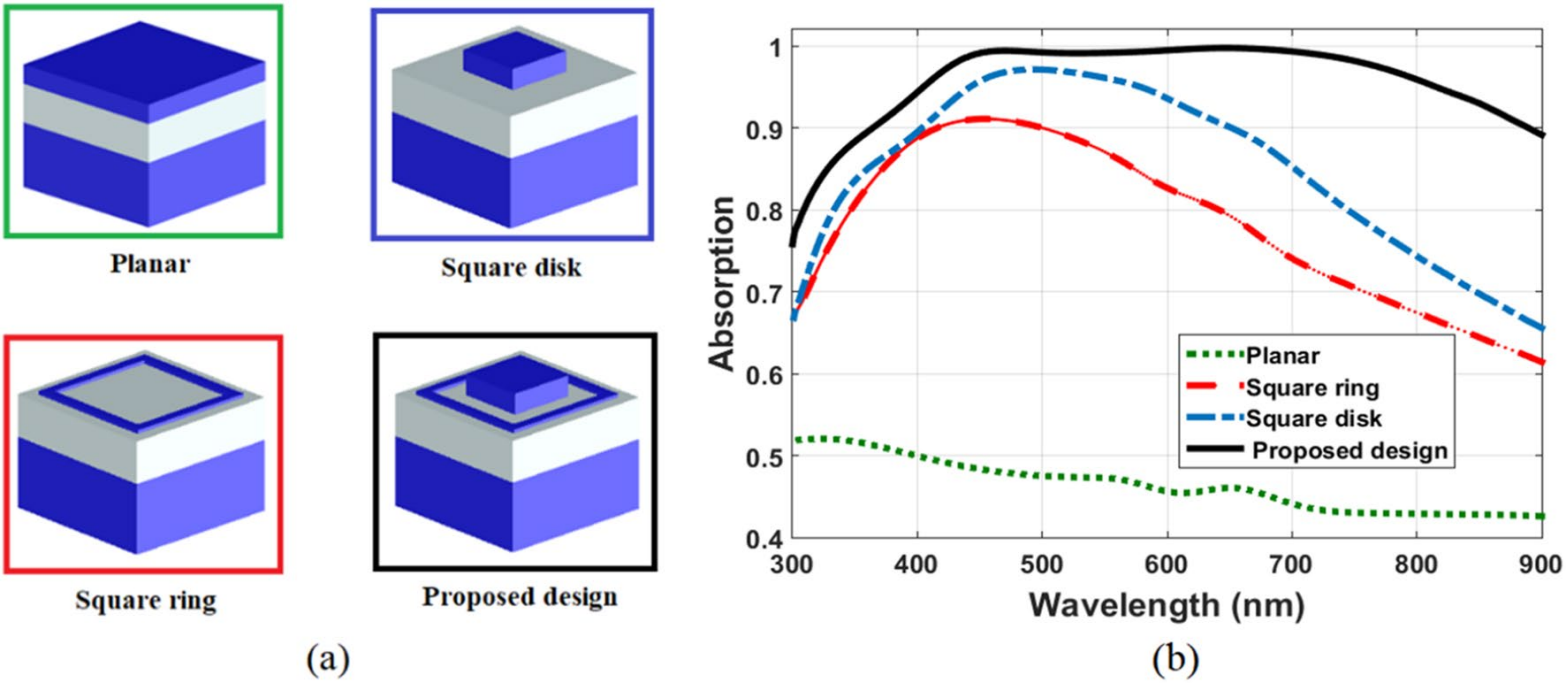
(**a**) a 3-D view of three different absorber configurations with the proposed design, and (**b**) Absorption spectrum for all three absorber configurations with the proposed one.

In addition, a comparison between absorption spectrum of the proposed MA with three other different configurations of the top layer resonators is investigated. The first configuration is a planar MIM continuous film in Fig. 5a which is performed to verify benefits of resonance modes. The other two configurations are a square ring and a square disk resonators. The layer thicknesses in all three configurations are consistent with the proposed design, and a single square disk with a length of 100 nm is utilized. Figure 5b displays absorption spectrum of the three configurations with our optimized absorber structure. For the planar MIM continuous film, the absorption of incident waves can be ascribed to the Fabry Perot cavity and the intrinsic loss of Mn. However, the absorption of the other two configurations (square ring and square disk resonators) are significantly increased due to plasmonic resonances. For our optimized absorber, designing a structure with two resonators in the unit cell can easily offer strong surface plasmon resonances. As previously demonstrated, strong interactions are generated with incident waves and excite strong plasmon (SPP, PSP and LSP) resonances. Therefore, the presence of both a square disk and a square ring resonators results in perfect absorption.

**Figure 6:**
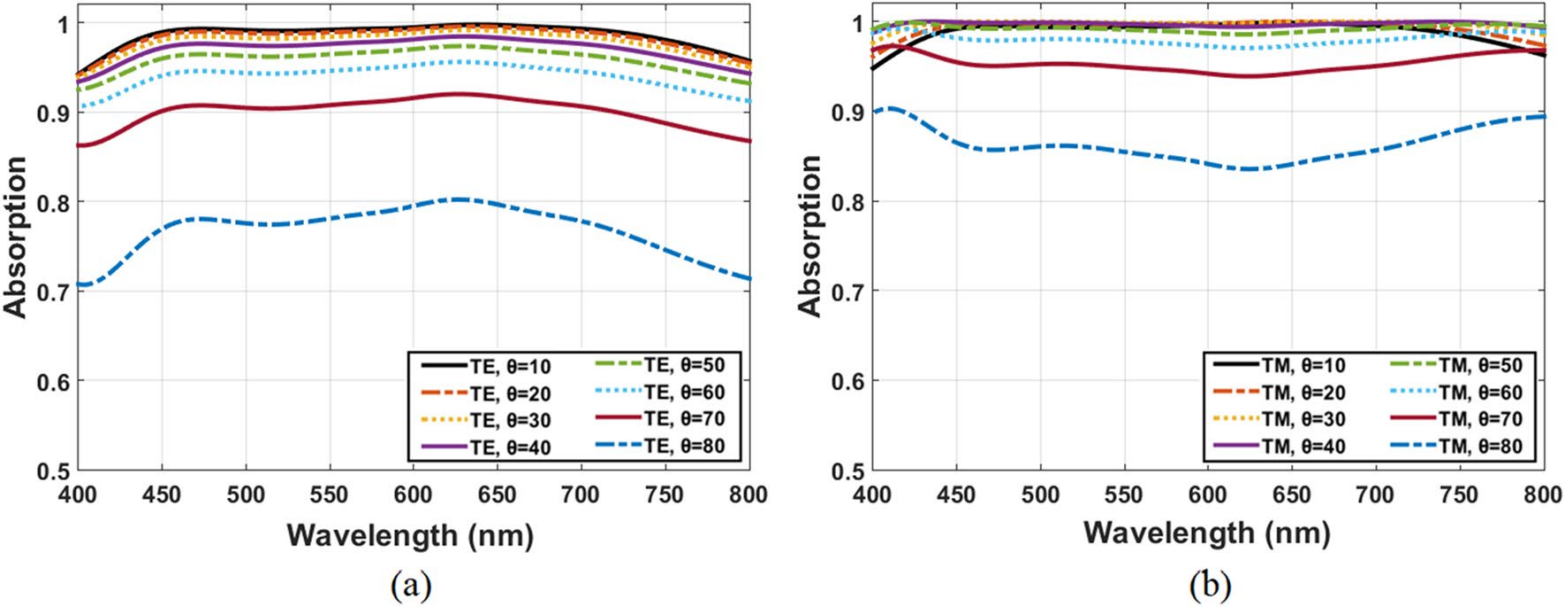
The structure absorption with incidence angle variation for (**a**) TE and (**b**) TM polarization.

An excellent absorber should be as robust as possible to changes in the source incident angle (θ). As a result, the absorber design is subjected to further simulation for oblique incidence with both TE and TM polarization in order to determine the variations in absorption. The absorption spectrum under oblique incidence is seen in Fig. 6a,b With TE and TM polarization respectively. Owing to the symmetrical geometry of the structure, it absorbs incident waves with more than 85% regardless of polarization at oblique incidence up to 70°. However, absorption decreased significantly at incident angles greater than 70° due to the sensitivity of resonant natures to polarization and incident angle. Most of earlier works test absorber polarization at level of 70% absorptivity as illustrated in the Table 2 of comparison. Therefore, the absorption curves exhibit large incident angle insensitivity up to 80° under 70% absorptivity for both TE and TM polarizations, primarily because of the high symmetry^39,40^. That is one of the advantages of the proposed absorber design.

**Table 2:** Comparison of earlier work with the proposed absorber design for visible regime.

References	Device configuration	Bandwidth range (nm)		Absorption (%)	Dimensions (nm)	Used materials	Polarization independent and angle (A > 70%)
		From	To				
^41^	Multilayer	475	785	> 80	160 × 160 × 400	Au, Si	Yes, θ ≤ 65°
^42^	Monolayer	400	650	> 90	410 × 205 × 380	Ni	Yes, θ ≤ 60°
^43^	Multilayer	400	700	> 80	800 × 800 × 700	SiO_2_, Ni	Yes, θ ≤ 70°
^44^	Two layers	262	709	> 90	240 × 240 × 170	Au, Si	Yes, θ ≤ 50°
^27^	MIM tri-layer	450	600	> 90	400 × 400 × 130	Al, SiO_2_	–
^28^	MIM tri-layer	389	697	> 90	1000 × 1000 × 225	W, SiO_2_	Yes, θ ≤ 60°
^29^	MIM tri-layer	400	750	> 80	200 × 200 × 65	W, SiO_2_	Yes, θ ≤ 40°
^9^	MIM tri-layer	400	750	> 80	200 × 200 × 135	W, SiO_2_	Yes, θ ≤ 50°
^45^	MIM tri-layer	380	765	> 90	380 × 380 × 180	W, SiO_2_	Yes, θ ≤ 70°
Our design	MIM tri-layer	400	800	> 94	300 × 300 × 190	Mn, SiO_2_	Yes, θ ≤ 80°

**Figure 7:**
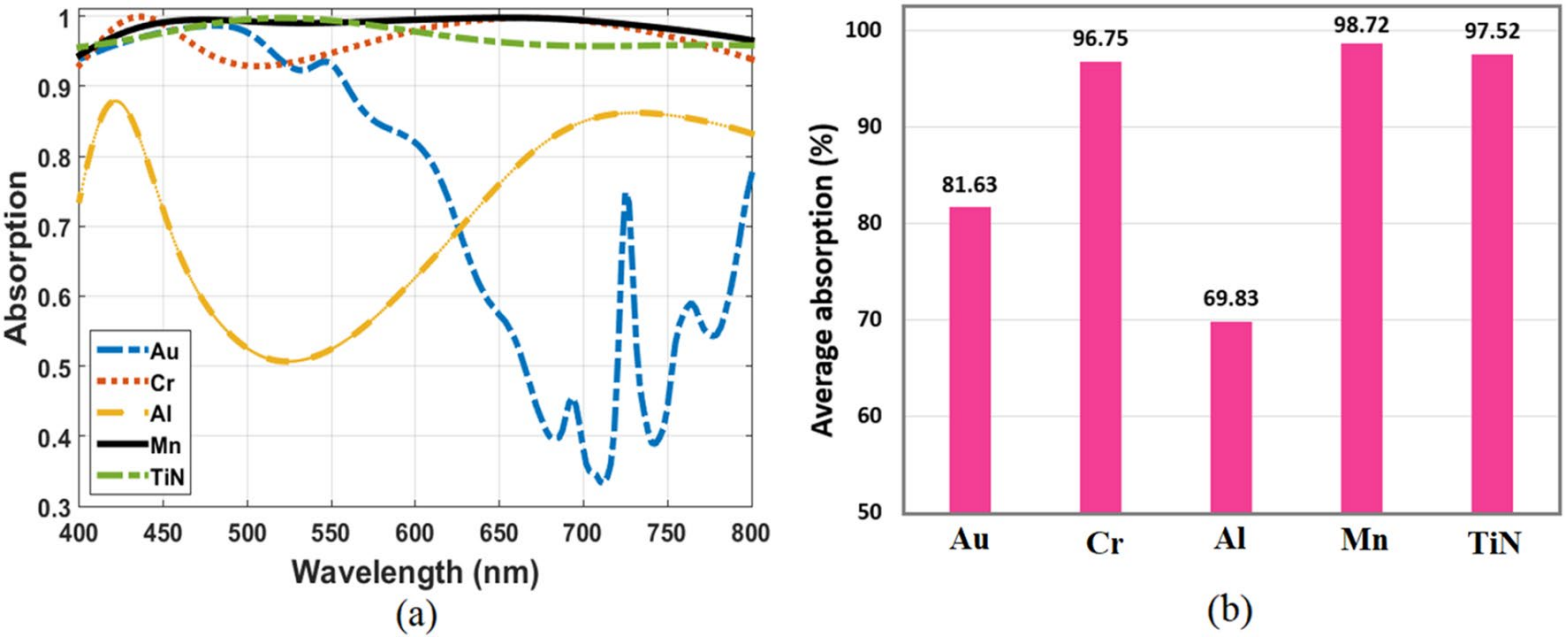
(**a**) Absorption spectra of the optimized structure with different metals and (**b**) Comparison of their average absorption.

**Figure 8:**
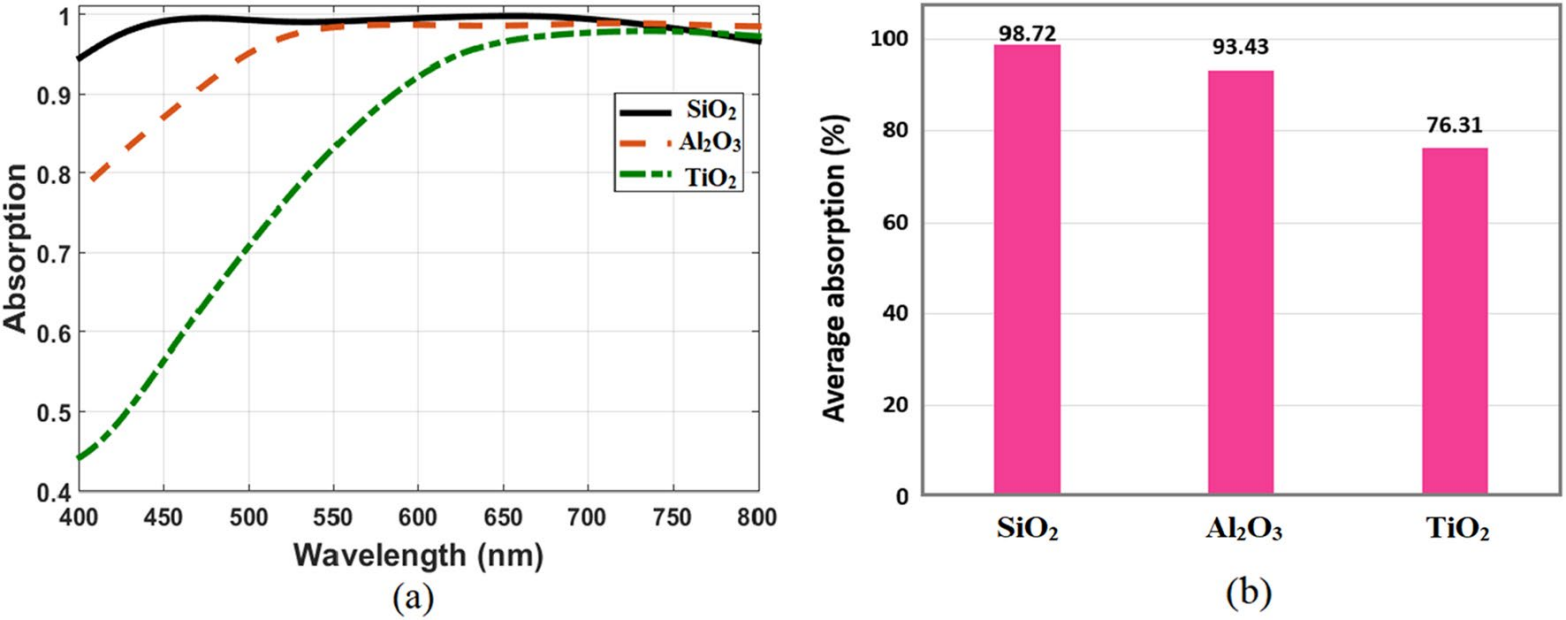
(**a**) Absorption spectra of the optimized structure with different dielectric materials and (**b**) comparison of average absorption.

Finally, the impact of using different metals and dielectrics on structure absorption is investigated. By replacing Mn from the top layer resonator and ground plane with different metals without changing any other dimensions. The response of the optimized absorber comprised of different metals such as Au, Cr, Al, and TiN is plotted in Fig. 7a. It is clearly observed that the absorption performance of metals such as Au and Al, suffers significantly. Even though when compared with absorber based on refractory metals such as Cr and TiN, it might accomplish significantly higher absorption. The reason of the behavior can be attributed to the wavelength-dependent refractive index of each metal. The plot also indicates that Mn based absorber is the best optimized for the proposed absorber because of the structure impedance matched to that of free space. The average absorption values of the stated metals are shown in Fig. 7b for the wavelength range of 400–800 nm. This figure implies that it is possible to replace Mn metal with TiN refractory metal while the average absorption is still high. However, the TiN-based absorber exhibits lower absorption spectra when compared to Mn. Figure 8 demonstrates the absorption spectra of the proposed absorber for different dielectric materials. The absorber is simulated with three dielectrics of SiO_2_, Al_2_O_3_, and TiO_2_, while the dimensions of the structure and the metal used (Mn) are kept the same. It is shown that the absorption shifted toward longer wavelengths as the refractive index of the dielectric material increased. Therefore, the dielectric of SiO_2_ has more contribution to the high average absorption than other dielectrics.

The comparison of earlier designs for visible band is presented in Table 2, with an emphasis placed on familiar characteristics such as absorption bandwidth, dimensions, material used, polarization independent and angle of incidence. It can be easily noticed that the proposed design covered all visible band compared with previous work with perfect absorption level. Broadband absorbers that can work through full region of visible band are relatively rare. Moreover, a perfect broadband absorber, with 98.72% average absorption and a resonance absorbance of 99.8% was found in our design. In addition to wide-angle sensibility at both TE and TM polarization up to 80° compared with earlier works at absorption level of 70%. The production costs are directly related to the materials used as well as the number of layers included. This design is unique in terms of thin structure, low material cost and easy fabrication in addition to high performance when compared to those stated in Table 2.

## Conclusion

Broadband perfect metamaterial absorber for visible band from 400 to 800 nm numerically demonstrated. This absorber is constructed by Mn–SiO_2_–Mn three-layer structure, with 190 nm thickness. Selection of the geometrical parameters values of the absorber unit cell is based on Particle Swarm Optimization (PSO) algorithm. As a result of thickness optimization, the proposed metamaterial absorber has 98.72% average absorption over the wavelength range of 400–800 nm, and achieves perfect absorbance (above 99%) over the wavelength range of 447–717 nm. Moreover, the absorption still remains beyond 85% when the angle of incidence changes from normal up to 70° for both TE and TM polarization. Based on these characteristics, the proposed metamaterial absorber structure is an excellent choice for visible light applications such as optical sensors, thermal emitters, and color imaging.

## Data Availability

The data sets used and/or analyzed during the current study are available from the corresponding author on reasonable request.
